# L'Hôpital de la Charité

**Published:** 1832-02

**Authors:** 


### HOSPITAL REPORTS.

### L'HÔPITAL DE LA CHARITÉ.

### *Laryngotomy and Tracheotomy performed upon a Patient attacked with Œdematous Angina, after the Use of Fumigations of the Deuto-chlorate of Mercury: Death during the Operation.**

A LABOURING man, twenty-four years of age, of good constitution, was admitted in the beginning of December, under Professor FOUQUIER. This young man had contracted syphilis in the summer of 1830, and had been very ineffectually treated: every symptom of the disease, however, disappeared. Some months before his admission, he was attacked with pain in the throat, difficult deglutition, hoarseness, weakness, and ultimately loss of voice. When he entered the Charité, he had an ulceration on the velum pendulum palati and uvula, with enlarged tonsils: it was presumed, from the change of his voice, that similar ulcerations existed in the upper part of the aerial tube. An anti-syphilitic treatment was adopted: he took, at first, mercurial pills, and afterwards fumigations of the deuto-chlorate of mercury were employed. After the first fumigation, he felt a pricking sensation in the larynx, and great dryness of the throat: still the fumigations were continued. About forty-eight hours after the first fumigation, he was attacked with dyspnœa, and those other symptoms which mark the existence of œdema of the glottis: he felt as if there was some foreign body in the throat, which impeded his respiration; his countenance expressed the greatest anxiety and suffering; he inspired with the utmost difficulty; the expiration was more easy; he complained of no pain in the larynx; the pulse was rapid, and there was imminent danger of suffocation.

The man having continued in this state for twenty-four hours, M. ROUX was requested to see him. For the purpose of determining the existence of œdema of the glottis, M. R. passed his finger to the base of the tongue, but he could not discover the soft tumor which is to be detected in such cases: at this moment, also, no serious accident was threatened, as the symptoms had undergone a decided remission.

On the next day (December 14th,) the dyspnœa was very severe, and all the symptoms were aggravated. M. Roux was again consulted. As the pulse was full and quick, some blood was taken from the arm, and in a quarter of an hour the patient was carried to the operating theatre, and M. Roux proceeded to perform the operation of laryngotomy: he had scarcely divided the crico-thyroidean membrane, when the patient made some attempts [illegible] expire, which caused the expulsion of a little frothy blood [illegible] denly the breathing was entirely suspended, the acti[illegible]

on of the [illegible]

* La Lancette Française.

R

heart ceased, the face became pale, and the patient died. M. Roux, believing that there existed some obstacle to the passage of the air at the lower part of the larynx, immediately performed the operation of tracheotomy. Air was blown into the lungs by means of a tube introduced into the opening made in the trachea: the lips of the patient were slightly moved, but respiration could not be re-established: it was evident that asphyxia had occurred, in consequence of the introduction of blood into the bronchia.*

Upon dissection, the larynx was found in the following condition: The edges of the epiglottis were eroded, and the cartilage was twice its natural thickness; upon its mucous membrane were numerous fleshy excrescences; the glottis was œdematous; the laryngeal opening was quite natural. Rather a deep ulceration was discovered upon a level with the vocal chord, on the right side. The lungs were gorged with blood, and presented all the appearances of a case of asphyxia.

* The Editor of La Lançette is rather inclined to believe that the death of the patient arose from the entrance of air into a vein during the operation, than from asphyxia produced by the admission of blood into the bronchia: he observes, that blood has frequently passed into the bronchia during the operation of laryngotomy, and has only occasioned efforts to expectorate and some threatenings of suffocation; and when the patient is too weak to get rid of the blood thus effused, or it cannot be quickly removed, death does not take place quickly, or without more vehement struggles than are described in the above; these effects are rather observed after the introduction of air into the vessels.—Vide *London Med. and Phys. Journal*, p. 122 and 175, vol. x.

